# The Microbiota–Gut–Brain Axis–Heart Shunt Part II: Prosaic Foods and the Brain–Heart Connection in Alzheimer Disease

**DOI:** 10.3390/microorganisms8040493

**Published:** 2020-03-31

**Authors:** Mark Obrenovich, Shams Tabrez, Bushra Siddiqui, Benjamin McCloskey, George Perry

**Affiliations:** 1 Research Service, Louis Stokes Cleveland, Department of Veteran’s Affairs Medical Center, Cleveland, OH 44106, USA; 2 Department of Chemistry, Case Western Reserve University, Cleveland, OH 44106, USA; 3 The Gilgamesh Foundation for Medical Science and Research, Cleveland, OH 44116, USA; ben@engineindustries.com; 4 Department of Medicinal and Biological Chemistry, College of Pharmacy and Pharmaceutical Sciences, University of Toledo, Toledo, OH 43606, USA; 5 Departments of Chemistry and Biological and Environmental Sciences, Cleveland State University, Cleveland, OH 44115, USA; 6 King Fahd Medical Research Center, King Abdulaziz University, Jeddah 21589, Saudi Arabia; shamstabrez1@gmail.com; 7 Department of Medical Laboratory Technology, Faculty of Applied Medical Sciences, King Abdulaziz University, Jeddah 21589, Saudi Arabia; 8 North East Ohio College of Medicine, Rootstown, OH 44272, USA; bsiddiqui@neomed.edu; 9 Department of Biology, University of Texas at San Antonio, San Antonio, TX 78249, USA; perry2500@gmail.com

**Keywords:** French paradox, polyphenol, cerebrovascular, heart, brain, microbiota-gut-brain axis, trimethylamine-*N*-oxide (TMANO/TMAO), co-metabolism, Alzheimer disease, Parkinson’s disease, prosaic foods, red wine

## Abstract

There is a strong cerebrovascular component to brain aging, Alzheimer disease, and vascular dementia. Foods, common drugs, and the polyphenolic compounds contained in wine modulate health both directly and through the gut microbiota. This observation and novel findings centered on nutrition, biochemistry, and metabolism, as well as the newer insights we gain into the microbiota-gut-brain axis, now lead us to propose a shunt to this classic triad, which involves the heart and cerebrovascular systems. The French paradox and prosaic foods, as they relate to the microbiota-gut-brain axis and neurodegenerative diseases, are discussed in this manuscript, which is the second part of a two-part series of concept papers addressing the notion that the microbiota and host liver metabolism all play roles in brain and heart health.

## 1. Introduction

We know that what is good for the heart is also good for the brain, most likely involving the shared cerebrovascular system. It is well established that a vegetarian and polyphenol-rich diet, including fruits, vegetables, teas, juices, wine, indigestible fiber, and whole grains, provides health-promoting phytochemicals and phytonutrients that are beneficial for the heart and brain. What is not well characterized is the effect these foods have when co-metabolized within our dynamic gut and its colonizing flora. The concept of a heart shunt within the microbiota-gut-brain axis underscores the close association between brain and heart health, and the so-called “French paradox” offers clues for understanding neurodegenerative and cerebrovascular diseases. Moreover, oxidation–reduction reactions and redox properties of so-called brain- and heart-protective foods are underappreciated with respect to their enhanced or deleterious mechanisms of action. Focusing on prodromal stages, and the common mechanisms underlying heart, cerebrovascular, and neurodegenerative diseases, we may unmask an understanding of the means to better treat these related diseases. Part I, in a two-part series of concept papers, focuses on novel findings involving nutrition, biochemistry, microbiology, and metabolism to suggest a heart shunt within the microbiota-gut-brain axis. Here, we explore the bacterial species that contribute to disease and health; we suggest that oxidative and inflammatory mechanisms drive heart and brain disease, and that wine polyphenols help protect us from bacterial-derived deleterious metabolites. Exploring aspects of co-metabolism within the microbiota-gut-brain axis and metabolites from prosaic foods could lead to a unifying hypothesis for age-related diseases and advance our understanding of vascular dementia, neurodegeneration, and heart disease. To simplify our understanding of the two most common and extremely complex diseases in the world today, namely, coronary heart disease (CHD) and Alzheimer disease (AD), we describe a shunt between the heart and the brain, where commensal microorganisms though the microbiota-gut-brain (MGB) axis do affect both the heart and the brain, which are coupled through the vasculature, and oxidative and inflammatory stressors are key propagators of disease pathogenesis for the brain and the heart.

There are many competing theories surrounding the pathogenesis of Alzheimer disease and other neurologic degenerative states. Unfortunately, little is accomplished in regard to curbing age-related diseases and neuronal degeneration. Several alternative hypotheses that do not support or refute current dogma are not mutually exclusive either. Alois Alzheimer described neuronal changes in a multifactorial, memory-robbing, and complex disease, which we call Alzheimer disease. However, he did not suffer from the illness and this is why it is not apostrophized in the medical nomenclature system. The disease itself has early manifestations and later ones as well, depending on the particular protein involved or the underlying genetic constitution of affected individuals. AD is characterized by hallmark lesions, namely, extracellular amyloid deposits or plaques and intracellular microtubule-associated proteins called tau within neurofibrillary inclusions or tangles. When we say that amyloid plaques and neurofibrillary tangles cause the disease, we look at end-stage lesions to make that statement. While correlation does not imply causation, it does offer clues into the pathogenesis of the disease. Answers to the pathogenesis of AD and many neurodegenerative diseases lie in understanding that they are largely a result of a protracted process, which many believe begins decades before any prodromal stages or pathology become evident. Our efforts to directly target extracellular amyloid deposits or the intracellular neurofibrillary tangles does not cure the disease nor does it give effective treatments or patient and care-giver relief. While plaques and inclusions are not benign, they are hallmark end-stage lesions, and we must search ahead of prodromal changes if we expect to develop a cure. The Alzheimer Association estimates that five million Americans live with AD in all its forms and, in the United States, every 66 s, a new case develops on their website. This number is expected to rise to as high as 16 million by 2050, since AD is an age-related disease.

Because the current hypotheses do not address the full scope of the disease, and due to lower funding levels, particular for young investigators, we made little progress in efforts to cure or halt the disease through drugs and treatments. Scientists work diligently to find means to not only treat AD but prevent it from developing in the first place. Unfortunately, there is little success in the approaches that modern science undertakes. Over the last four decades, we explored the lesions involved but did not fully address the underpinnings or precipitating events and mechanisms. New and growing evidence links the trillions of microbes that inhabit the human gastrointestinal tract to neurological conditions and diseases. What is good for the heart is good for the brain, and the concept of a heart shunt within the microbiota-gut-brain axis underscores the close association between brain and heart health. Moreover, oxidation–reduction reactions and redox properties of so-called brain and heart-protective foods are underappreciated, as are any enhanced or deleterious mechanisms of action involving such small molecular compounds.

Previously, we reported a commensal co-metabolic relationship between the microbiota and the host [1,2,3]. Some consider only symbionts to be the holobiome; however, when we discuss the microbiota, we consider all microorganisms as a microbiome and include all bionts within a host. This relationship goes well beyond simple nutrition and metabolism and includes the prospect for bugs as drugs, as we call them. Furthermore, in the case of mental and brain disease, we include all microorganismal bionts, which engage as generators of beneficial co-metabolism, to be psychosymbiotics. This powerful concept will one day change our interpretation of lab results, patient histories, and genomic testing, and it will become a key component of what will be truly known as personalized medicine. We propose that this novel concept will lead to better treatment, diagnostic, and management approaches, which could one day prevent disease or, conversely, contribute to research and understanding of disease pathobiology [1].

**Figure 1:**
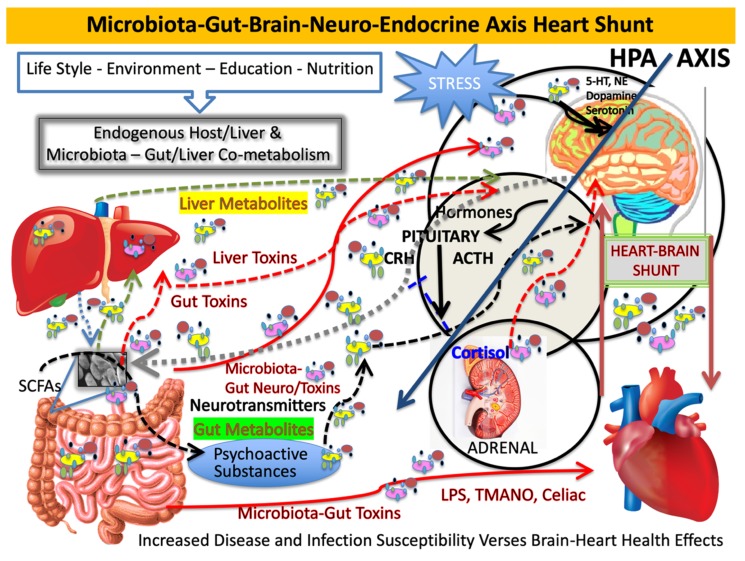
Action of microbiota through hypothalamic–pituitary–adrenal (HPA) axis and brain via chemical messengers. Depicted here is the microbiota-gut-brain axis–heart shunt and the intersection between the gut-derived messengers vs. liver and bacterial toxins or metabolites and the effect that stress and short-chain fatty acids (SCFAs) have on the system. This can arise form prosaic foods, drug action, or co-metabolism between the microbiota and the host.

Our microbiome affects the microbiota-gut-brain axis both directly and indirectly. Multiple routes of gut–brain communication are established. One pathway is through neural networks that control the enteric nervous system independently or through the central nervous system (CNS) via sympathetic efferent prevertebral ganglia. Others include parasympathetic efferent nerve signaling through the vagus nerve [2]. It is at these nodes where the microbiota has indirect action on the hypothalamic–pituitary–adrenal (HPA) axis and brain through chemical messengers acting as neurotransmitters and secondary messengers [3] (see Figure 1). We are currently classifying small molecules from the microbiota, which originate distal to the brain and yet reach brain compartments. This happens via diverse indirect routes like the vagus nerve, as well as directly and systemically when the gut-derived or liver-modified compounds cross immune barriers to enter the brain and other immune-privileged compartments [4]. Whether these compounds are beneficial or deleterious, they need to reach a level that possibly can affect the brain to result in physiologic changes. We now suggest a new pathway for the MGB axis, which we now consider to be coupled, namely, the microbiota-gut-brain–heart shunt. In simplest terms, the shunt is any connection or opening or coupling of systems and organs that allows for movement within or from one part of the body to another. Shunting, in biochemical terms, is usually bidirectional and reversible. For example, enzymatic processes and enzymes, like transketolase, which drives the hexose monophosphate shunt, are analogous to the MGB axis, and flux depends on precursor levels or mass action. When shunts act similarly to anatomic shunting, such as congenital heart defects or when used to bypass organs or other systems, shunting is usually directional. Nevertheless, we describe a shunt that affects metabolite flux, which is easily shared between the coupled systems and has similar effects on both conjoined organs.

In describing the tight coupling between the heart and the brain, Obrenovich and colleagues found common AD and heart protein expression that led to the development of a unifying theory for AD, which helped explain cerebrovascular aspects of the most common neurodegenerative disease. AD has components of dementia, as well as cognitive and neuropsychiatric manifestations. Neural communication pathways within the enteric nervous system, a main division of the autonomic nervous system, affect gastrointestinal function and are coupled with the vagal afferent nerves that transmit sensory information from the visceral organs to the CNS. Thus, the gut is a major communication route to the brainstem and CNS, and the vast majority of vagal nerve fibers are afferent [5], which sense regulatory gut signals and peptides, such as leptin, ghrelin, and others [3]. Nevertheless, we know that what is good for the heart is also good for the brain, most likely involving the shared cerebrovascular system, as in a shunt. It is well established that a vegetarian and polyphenol-rich diet, including fruits, vegetables, teas, juices, wine, indigestible fiber, and whole grains, provides health-promoting phytochemicals and phytonutrients that are beneficial for the heart and brain. What is not well characterized is the effect these foods have when co-metabolized within our dynamic gut and its colonizing flora.

## 2. Modulating AD through Prosaic Foods and Drugs

When one considers ways to affect and modulate one’s own health, one must start with diet and lifestyle. Beyond our immediate dietary choices and variable consortium of gut bacteria with which we are colonized, higher education and exercise positively affect and modulate the risk and progression of AD. Furthermore, oxidative and inflammatory stressors also modulate AD and many age-related disease risks. Thus, antioxidant foods and anti-inflammatory agents are expected to provide the most impact. As it turns out, other common things also do this, including prosaic items such as wine, tea (phenolic acids), some spices, select nonsteroidal anti-inflammatory drugs (NSAIDS), statins, some antidepressants, nootropics [6], and euglycemic medications or insulin sensitizers [7] to name a few. Other attempts to improve function targeted cellular mechanisms and neuronal stimulation to treat or delay AD. These include things such as caffeine, nicotine, nootropics, other psychostimulants, vasodilators, and opiate antagonists [8]. Nootropics, so-called smart drugs, display anti-hypoxic and anti-amnestic effects, prevent memory impairment, and facilitate cognitive function, which we know occurs with brain aging, insult, trauma, and in various diseases. This happens, in part, through both stimulatory and cholinergic mechanisms.

For Alzheimer research, the French paradox is an interesting consideration. There is not yet a clear mechanism for how the French enjoy high-fat foods with heart disease-sparing mechanism, and more work is necessary to address these aspects. Taken together, we argue that oxidative stress through microbial action and metabolism could contribute to trimethylamine-*N*-oxide (TMANO/TMAO) formation, but other factors may be more important in modulating heart and cerebrovascular disease forms of AD or vascular dementia (VaD) (see Figure 1 for an overview). Prosaic foods, such as red wine, its constituents, or its co-metabolized chemicals, have intriguing properties that include acting on endothelial cells and the cerebrovascular system [9]. Endothelin-1 (ET-1) is important in AD, since it is a highly potent peptide and vasoconstrictor [10]. It is associated with atherosclerosis, and we showed that ET-1 has a strong relationship with AD pathogenesis and cytokine stress [11], while high expression of ET-1 is known to contribute to the development of cerebrovascular disease, AD, and cancer. Prosaic foods, including red wine, were shown to inhibit ET-1 synthesis. Conversely, prosaic foods, such as eggs and those containing phosphatidylcholine (PC), are associated with CHD but this does not involve the cholesterol theory. Rather, it involves absorption, where host enzymes convert trimethylamine (TMA) to trimethylamine-*N*-oxide [12]. TMANO formation in humans is poorly understood but may contribute to bacterial pathobiology of clot formation. This illustrates a well-supported notion of a heart–brain shunt, and it allows exploring the microbiota-gut-brain axis–heart shunt, whereby microbe-derived metabolites not only reach the brain and brain compartments but also have an effect on the heart. Incredibly powerful, this supports our premise and demands a call for greater funding levels if more progress is expected to happen with AD research.

All of the aforementioned prosaic drugs and foods show efficacy in both treating and delaying AD or they helped to elucidate its pathogenesis. For instance, diabetes has an incredibly adverse effect on the heart and brain. Recently, a newer form of AD, called type 3 diabetes, was described. Using diabetes drugs, such as the insulin sensitizer metformin, which is suggested to have anti-aging and many other positive health effects, could modulate AD and CHD risk. In diabetic mice, metformin corrected memory impairment and abnormal transport of amyloid beta (Aβ) across the blood–brain barrier (BBB) [13]. One human study, exploring the microbiota and metformin, compared administration in both diabetic and non-diabetic individuals. The diabetics were found to have higher abundance of a mucin-degrading microbe *Akkermansia* species. When the researchers pooled short-chain fatty acid (SCFA)-producing and mucin-degrading microbes, they found that *A. muciniphila* and *A. butyrivibrio* were more abundant in the gut of type 2 diabetics on metformin than in those diabetic cohorts not on metformin. The presence of a higher number of colony-forming units of both types of bacteria suggests that metformin seems to improve the integrity of the intestinal mucosal barrier, favoring colonization of these species, and it explains the improved mucin layer maintenance and lower inflammation by reducing any translocation of proinflammatory lipopolysaccharide (LPS). This could aid in controlling adipose tissue metabolism and fat storage, as well as help improve glucose homeostasis, but perhaps only in diabetics [14]. This group recapitulated findings with aging and antibiotic use, which offers an explanation for reduced alpha diversity and the growth of opportunistic pathogens, such as species of *Escherichia* and *Shigella*. Nevertheless, metformin is the most commonly prescribed drug for type 2 diabetes and is considered quite safe when administered to healthy adults. Its putative neuroprotective properties, when taken with meals, could reduce hyperinsulinemia by downregulating insulin-like growth factor-binding protein and increasing free insulin-like growth factor-1 (IGF-1) [15].

AD has both an oxidative stress component and a neuro-inflammatory component to its pathogenesis. Amyloid beta (Aβ) fragments like Aβ 1–42 form insoluble aggregates that are toxic, whereas others are relatively harmless. When it comes to AD, anti-inflammatories like ibuprofen, aspirin, and naproxen sodium, which lower inflammation, should reduce the risk of developing AD through the inflammasome mechanism. However, with NSAID studies, it was found that they affect the production of Aβ through changing the cleavage site for amyloid precursor protein (APP) in nerve cells. Alpha, beta, and gamma secretases are the enzymes responsible for the differential production of amyloid fibrils of various lengths through this cleavage mechanism. However, it was shown that amyloid causes the microglia to release pro-inflammatory cytokines like interleukin (IL) 1β and tumor necrosis factor (TNF) alpha, among others [16]. NSAIDs are believed to increase the favorable cleavage of APP by directing APP–gamma secretase, an enzyme complex with presenillin1 (PS1), and reshaping the protein cleavage site, thereby reducing Aβ 1–42. NSAIDs do not reduce the overall quantity of Aβ molecules but change the PS1 confirmation, shifting APP toward shorter fragments like Aβ 1–38 [17]. Unfortunately, trials were halted due to cardiovascular problems observed with frequent use of many NSAIDS [17]. It is possible that NSAIDs, many of which contain one or more benzene rings and food-derived phenols, when processed or co-metabolized with gut bacteria, could also affect APP cleavage.

Importantly, amyloid in all of its forms has an unknown function, but it is known to bind toxins, metals, and compounds through a bioflocculant or precipitation mechanism, resulting in phagocytic presentation to microglia for clearance [18]. This mechanism is similar to salivary proline-rich proteins, particularly the basic ones, which form insoluble complexes with compounds like condensed tannins and lignans, thereby also acting effectively as a type of bioflocculant. Moreover, in AD, iron and copper distribution is altered [19], and amyloid binds these divalent cations directly and, depending on the metal species bound, either propagates or dismutate oxidative stress by quenching hydroxyl free radical activity [18]. Metal-catalyzed oxidation is involved in aging, in age-related diseases, in aerobic metabolism, and in diabetic complications. These include glyocoxidative stress (oxidative stress coupled with glycation), one of the many redox-active mechanisms that propagate protein, lipid, and nucleic acid damage [20,21,22]. Furthermore, Aβ was recently recognized as part of the innate immune system as an antimicrobial peptide [23,24]. This finding both supports the bioflocculant concept and hypothesis for Aβ protection and illustrates that microbes and viruses could be responsible for initiating AD pathogenesis long after the initial infection or occurrence and after precipitating events resolve. It is important to note that not all bacteria are harmful. Intrinsically, empirically, and by definition, commensal and pathogenic bacteria have very clear differences, and an important difference is their localization when breaching barriers or breaching host defenses [25].

This brings us to the point of a leaky gut, celiac disease (CD), and the possible downstream consequence of a leaky gut begetting a leaky brain [25]. Growing attention is focused on the involvement of the gut–brain axis and microbiota dysbiosis in CD, which is commonly known as a systemic disorder with multifactorial pathogenesis and multifaceted symptomatology. CD, so-called “celiac brain”, is now shedding light onto the fast-growing importance of microbiota dysbiosis and its involvement in brain function. In an attempt to assess neurological changes such as so-called “brain fog” and dementia, which are known to occur in adult celiac patients, transcranial magnetic stimulation (TMS), a non-invasive neurophysiological technique, is now being used to assess and monitor such neurological changes [26].

In this framework, TMS studies carried out so far in CD patients seem to converge on an impaired central motor conductivity and a hyperexcitable celiac brain to TMS, which partially reverts after a long-term gluten restriction [27]. Notably, a clear hyperexcitability is a stably reported feature of both degenerative and vascular dementia, which also partially share an impairment of the central cholinergic pathways, now found to be more evident in AD and mildly cognitively impaired patients compared to those with vascular cognitive impairment [28]. Therefore, given its potential neuroprotective effect, assessment for gluten sensitivity or a gluten-free diet could be introduced in patients with celiac disease or sensitivities, although the overall response in neurological symptoms (and cognition in particular) is still controversial [26]. Nevertheless, identifying new and possibly modifiable risk factors, like gluten intolerance or gut sensitivity, may especially be of crucial importance to those patients with CD.

Gut dysbiosis seems to play a crucial role in depression and some mental disorders. Along with possible implications for the antimicrobial effects of antidepressant drugs, antidepressant effectiveness may be modulated by the microbiota [29]. An altered microbiota is linked to neuropsychological disorders, such as depression and autistic spectrum disorder (ASD). Gastrointestinal disorders like inflammatory bowel disease and irritable bowel syndrome are associated with mental disorders [30]. In combination with a leaky gut, a leaky brain could lead to potential negative neurological consequences, such as increased severity of depression, anxiety, or a host of other neurological diseases [25]. Antibiotic interventions are very disruptive for the gut flora and often lead to dysbiosis, which can impact the metabolites produced by the gut microbiota [31,32]. This could ultimately have negative consequences for patients with an infection and also for those who have depression or anxiety due to alterations in gut-produced metabolites or hormones [33]. Therefore, new methods are needed for treating patients, while protecting their microbiota, if antibiotics are to be used.

## 3. Oxidation–Reduction Reactions Involving the Brain and Mammalian Gut

Reactive oxygen (ROS) or nitrogen species (RNS) are implicated in oxidative stress and damage, which is a paradoxical component of aerobic metabolism [34]. Since we cannot escape oxidative metabolism, and oxidation is a very common enzymatic mechanism, one must consider oxidation–reduction pairs in every system explored. Oxidation–reduction or redox potential (Eh) is measured in millivolts and represents the affinity of electron transfer to or from a chemical species in solution. The ability to either gain or lose electrons in that particular environment is concentration- and temperature-dependent. Chemical species with higher, i.e., more positive, reduction potentials than a particular species introduced into the system tend to gain electrons from the new species and become themselves reduced by the oxidizing species, whereas solutions with lower, i.e., more negative, reduction potential tend to lose electrons to the new species and become themselves oxidized by reducing the new species [35].

The brain responds to distressful physiological stimuli like oxidative stress, infection, neuronal injury, or trauma through astrocyte and microglia activation, which then secrete a host of inflammatory mediators and cytokines. The author elucidated a downstream consequence in AD, whereby massive nitric oxide (NO) production, largely from expression of the inducible form of nitric oxide synthase (iNOS), contributes to pathogenesis in AD. NO is an important vasodilator but reacts rapidly with the superoxide anion to form the RNS peroxynitrite anion. Nitric oxide-dependent oxidative stress results in ultrastructural cellular alterations and damage to mitochondrial DNA in AD cases [11]. With a greater focus on oxidative stress, rather than on cholesterol alone, as the French paradox would suggest, colleagues at Case Western Reserve University USA elucidated how NO, which is secreted by gut bacteria, was able completely change nematodes’ ability to regulate their own gene expression. This study was the first to demonstrate how gut bacteria could affect ubiquitous mammalian NO networks and offer a possible mechanism in mammals, including humans [36]. Of course, the researchers did force the microbial mechanism by feeding NO-producing bacteria to the worms and, via mass action, they were able to affect DNA-silencing proteins and impair healthy worm development. We could extrapolate this to any fetus overexposed in utero to excess NO, or we could consider NO and reactive nitrogen free radicals as possibly being gut-derived, contributing to the RNS aspect of the disease. However, we must not ignore the many beneficial aspects of NO as a signaling molecule, when targeting this process.

Further support for an antioxidant hypothesis and mechanism for inhibiting bacterial-derived oxidation products comes from previous studies using selective mitochondrial antioxidants. To explore mechanisms for age-dependent protection on cerebral blood flow in apolipoprotein E (ApoE4) transgenic mice and in rats, administration of acetyl-l-Carnitine (ALCAR) and *R*-alpha-lipoic acid (LA) was performed [25]. Comparing ApoE4 mice vs. age-matched wild-type controls, ^14^C and iodinated anti-pyrene autoradiography demonstrated gradual brain hypoperfusion. Treatment with ALCAR plus LA was reported to prevent ischemic damage in rats and ApoE4 mice. Transmission electron microscopy showed ultrastructural damage in young and aged microvessel endothelia, perivascular cells, perivascular nerve terminals, hippocampal neurons, and glial cells, which coexist with mitochondrial structural alteration and mitochondrial DNA overproliferation and deletion in all brain cellular compartments. Temporal and spatial memory tests showed a trend in improving cognitive function when ALCAR antioxidant was used in combination. This suggests that a mitochondrial redox potential shift is important in brain protection with these compounds. Previous studies reported the abolishment of oxidative stress-induced structural changes in brain parenchymal cells (neurons, vascular wall cells, and glia) and restored cognitive performance by the same mitochondrial antioxidants, i.e., ALCAR + LA, in a two-vessel occlusion model of vascular dementia (VaD) aged rats [37,38].

More support for a bacterial role in redox mechanisms and diseases, possibly including ulcerative colitis, comes from the formation of hydrogen sulfide gas (H_2_S), which at neutral pH is ionized to sulfide anion [39]. High concentrations of H_2_S are correlated with sulfate-reducing bacteria, meat-rich diets, or the high fecal sulfide concentration derived from the bacterial degradation of cysteine, methionine, and sulfated gut-proteins [39]. H_2_S comes from oxidized sulfur-containing foods (meat or wine) and food additives, such as sulfite, sulfate, and sulfur dioxide, which was shown to inhibit butyrate oxidation in colonocytes [39]. The toxicity or protective aspect of hydrogen gas or H_2_S is not well understood, but the chemoprotective aspect of butyrate in the gut is well known. So is the danger and toxicity from heavy metals, such as mercury, lead, and arsenic, which are among the top 10 United States Department of Agriculture poisons and toxins due to frequent human exposure and the disease burden following acute and chronic exposure [13]. Among their common characteristics is the chemical affinity to proteins and non-protein thiols. Moreover, amines and intestinal bacteria are expected to react with nitrite to form nitrosamines and other nitrogen-containing toxic compounds, which are implicated in AD [25].

## 4. Statins, Other Natural Statin-Like Compounds, Small G-Proteins, and G-Protein Coupled Receptor and Cognate Kinase 2 (GRK2) in AD

Some statins, which are cholesterol-lowering drugs also show efficacy against AD, but by diverse means. In that regard, late-onset AD is associated with the ApoE gene involved in cholesterol and triglyceride transport and metabolism. Patients with one or more copies of the ApoE 4 allele are more likely to have Aβ aggregation and deposition and are more likely to get AD. Statins, like Lipitor (atorvastatin) and Zocor (simvastatin), also affect APP cleavage in ways similar to NSAIDs. This occurs through the Rho small guanosine triphosphate (GTP)-binding protein pathway and the activation of Rho-associated kinases (ROCK) 1, which promote the aberrant cleavage of APP and increase the production of Aβ 1–42 [40].

G-proteins in AD involve G-protein coupled receptor (GPCR) and cognate kinase (GRK) 2 activation, which Obrenovich and coworkers first identified in AD brains, observing its role in early AD pathobiology and before any prodromal stages. At that time, Obrenovich and colleagues postulated a brain–heart connection for AD pathogenesis as this kinase bridged our understanding of disease in both organs [41]. Others went on to include GRK5 in this process [42]. It is the overexpression of GRK2, and to a lesser extent GRK5, which the author, and others, found in the cytosolic fractions of hippocampal and cortical brain homogenates. This overexpression increased with age and with amyloid plaque deposition and began before any prodromal stages of AD were evident. This observation was also expected to occur in within microglia. We described the subcellular localization of GRK2 in neurons and the earlier involvement of vascular lesions in vivo as a key event in the neuropathogenesis and early development of human AD and AD-like pathology. Furthermore, we described the ectopic localization and expression of cell-cycle markers in AD [43], and we showed that GRK2 modulates cell-cycle progression [44].

Our understanding of the early activation of GPCRs, with their cognate kinases, is that they control many cellular functions, including cytoskeletal organization and post-translational modifications such as phosphorylation and gene expression, among others. Moreover, multiple drugs target GPCRs and some are likely neuroprotective. When researchers examined Rho, a small GTP-binding protein, and its pathway and activation in AD and APP cleavage in culture, they found that Rho activated ROCK1 [40] and perhaps ROCK2. Importantly, statins like Lipitor and Zocor can inhibit ROCK1 activity and favor good APP cleavage. This finding does not preclude an indirect mechanism, whereby ROCK1 phosphorylates an accessory molecule, which then interacts with alpha secretase. Our colleagues, when at Case Western Reserve University USA, noted that amyloid deposits activated pro-inflammatory cytokines and that statins inhibited amyloid-stimulated monocyte and microglial activation, as well as the subsequent release of pro-inflammatory cytokines like TNF-alpha and IL1-β, and they demonstrated a new function for statins other than or independent of their anti-inflammatory properties [45]. Since cholesterol could not reverse the suppression, it was concluded that the action was also independent of any cholesterol-lowering properties. Moreover, in rodent models, bacteria through the MGB do control neuroinflammation [46], and other aspects of brain inflammation in terms of motor deficits are being elucidated.

There is no question that cholesterol in the neuronal cell membrane influences the activity of secretases. Candidate AD drugs are also a cardiovascular disease drug targets, such as inhibitors of acyl-coenzyme A, cholesterol acyltransferase (ACAT), like Avasimibe, which regulates cholesterol distribution. Inhibiting ACAT lowers the production of Aβ. Kovacs and colleagues found that lowering cholesterol with ACAT inhibitors also improved learning in mice independent of cholesterol distribution [47]. Thus, it appears that what is good for the heart is also directly good for the brain, and the author maintained this for decades arguing for a strong cerebrovascular component to AD. Cholesterol fits into the French paradox story at several nodes, but microglia are central in this discussion, as NSAIDs and statins both share the APP cleavage mechanism, and aberrant cleavage is decreased when inflammation is suppressed in microglia.

## 5. Oligomers, Aβ-Derived Diffusible Ligands (ADDLS), Amyloid Fibrils, and Conformational Protein Changes

Amyloid beta plaque deposition does not correlate well with the severity of AD symptoms. Obrenovich, Perry, and coworkers speculated that Aβ is not in fact entirely toxic and has useful function, some even protective, and they argued that the protracted course of the disease has in fact an earlier mechanism of pathogenesis. Therefore, amyloid theorists began to further split the questionably toxic species Aβ into smaller species called Aβ-derived diffusible ligands (ADDLS) and amyloid oligomers [48]. The convention here is that not all fibrils are created equally, and different forms are differentially dangerous, such as proto-filaments of Aβ that have two isoforms, one toxic the other not. The resulting oligomers of the proto-filaments are suspected of being neurotoxic by configuration and conformational mechanisms, similar to prion disease pathogenesis in structure [49]. In that regard, Obrenovich and coworkers and others studied polyphenolic antioxidants and glycation mechanisms in diabetes and AD pathogenesis, suggesting that a protracted form of AD might be first initiated through a conformational change in protein structure [50,51]. Furthermore, a preventive mechanism to address confirmation changes, analogous to Congo red staining and birefringence, could hold the answer to protecting neurons from forming beta pleated sheet conformational changes in globular or other proteins and preventing subsequent glycation-mediated filamentous changes considered to be pathogenic.

In that regard, curcumin, tea, and other spices could bind to suspect proteins and either prevent conformational changes or suppress their toxicity, since these epitopes are suspected of mediating toxicity in conformational disease. Curcumin is an antioxidant that we speculated would be important for inhibiting or quenching oxidative stress in AD pathogenesis [50,52]. However, another beneficial mechanism of action could be at work here, as curcumin was shown to break up Aβ plaques in mouse brain models and differentially bind fibrillar amyloid forms in in vitro systems [53]. The curcumin studies that showed inhibition of Aβ and fibril formation were done in vitro, illustrating that absorption and brain delivery present hurdles to approaches using beta pleated sheet-binding substances. In that regard, curcumin and turmeric are difficult to absorb in dietary form and they do not cross the blood–brain barrier. Moreover, the stain and dye Congo red is most likely toxic to cells and, thus, its therapeutic use is questionable. Yet, other similar functioning compounds from food or co-metabolism may exist. New and safer compounds could be synthesized with this mechanism of action. The bioavailability of prosaic foods could also be improved or modified to cross immune barriers, thereby having an effect on aspects of conformational states or enhancing clearance of deleterious structural modifications suspected in precipitating disease pathogenesis. Efforts to improve bioavailability should be studied and proven before we can advocate supplementing or dosing spices and foods to treat disease. However, there is one study which demonstrated that adding curcumin to the mouse diet did break up Aβ plaques in rodent brains [54]. Unfortunately, it is unclear if this system mimics human physiology. While mice are the best current model we have to recapitulate human diseases, they often do not always mimic them fully or recapitulate all aspects of human disease. Nevertheless, when we consider dietary-derived phenomenon or mechanisms, we must then add in the microbiota aspect, as bacteria and/or microorganisms outnumber human cells by factors of 10 to 100-fold due to their abundance and size and ability to change biochemistry through mass action. These are contributing factors and potential sources for confounders, especially when findings involve transformation, modification, or formation of new compounds not derived from the host metabolism that could indeed reach brain compartments. Modified compounds possessing new beta pleated sheet-binding properties could perhaps offer potential clues about conformational disease mechanisms or explain variant and equivocal findings in this regard. When considering any natural product claim or mechanism for monitoring or treating diseases, like AD or other neurodegenerative diseases, one must exercise caution, while expecting purity and integrity, and we should insist on some clinical proof or peer-reviewed support from those monetizing supplements for these results in the production of unknown protective metabolites. Furthermore, whether the aim is to treat conformational disease, dissipate aberrant protein aggregates, or attenuate aberrant signaling, we must explore all possible contributions from enteric gut bacteria.

## 6. AD-Protective Foods and the Microbiota

Other protective mechanisms involving the aforementioned compounds, including curcumin and phenolic acids and their modification to smaller compounds, have many mechanisms of action, some of which may come from digestion or breakdown of these prosaic products by bacterial metabolism or when co-metabolized with the host. One thing needed in the discovery process is a battery of tests involving either fecal culture or select bacterial clones isolated and studied in regard to metabolism, building a new database for bacterial co-metabolism. This approach can serve to identify the chemical metabolic compounds within these digestive systems, and this may lead to new drugs, drug targets, and enzyme inhibitors. For example, in AD, a primary early degenerative process leads to the damage of cholinergic neurons in the brain, resulting in cognitive impairment. The level of neurotransmitter acetylcholine (ACh), a small molecule important in cognition and memory, is low in the brains of AD patients, and some speculate that reduced synthesis of acetylcholine in the brain is the cause of AD. However, dietary choline, phosphatidyl choline, lecithin, and betaine all can be converted into trimethylamine-*N*-oxide (TMANO/TMAO) via the metabolic enzymatic action of anaerobic bacteria and the liver [55]. Prior to absorption, dietary choline, a nutrient found largely in eggs, organs, and other animal foods, and carnitine are metabolized in the large intestine by gut microbiota to trimethylamine (TMA), as well as to dimethylamine and methylamine, albeit in lesser amounts [56]. After absorption, host enzymes convert TMA to TMANO in the liver via liver flavin monooxygenases [57].

One promising treatment approach is to enhance ACh levels by selectively targeting the enzyme which breaks down acetylcholine in the cholinergic synapses to acetate and choline. Acetylcholinesterase (AChE) is the enzyme largely responsible for the hydrolysis of ACh, but nonspecific cholinesterases hydrolyze ACh as well. One such enzyme, which is a plasma choline esterase, is the pseudo-cholinesterase known as butyryl cholinesterase (BChE). There are potent AChE drugs that are readily absorbed, such as AChE inhibitors esolerineare, phenserine, and tolserine [58,59]. While inhibiting the AChE enzymes could offer insight into protective mechanisms, nothing yet found is quite perfect. Nevertheless, several investigations reported that a reduction in AChE activity compensates for BChE activity, and they explored plant-derived phytochemicals for their AChE inhibitory activity. Natural compounds are of great interest in drug discovery. In that regard, natural cholinesterase AChE inhibitors lead the search for compounds for AD treatment, as they should have significant inhibitory activities against both AChE enzymes [60], but few studies reported the co-metabolism aspects surrounding acetyl cholinesterase inhibitory activity.

Many natural product compounds have AChE inhibitory activity. For example, resveratrol, quercetin, and curcumin all inhibit AChE and BChE. However, those products that inhibit AChE alone include the tea catechins, namely, the gallocatechins, such as epigallocatechin, epicatechin, epicatechin-3-gallate, and epigallocatechin-3-gallate, as well as the cinnamic acids and caffeic acid [60]. Moreover, nootropic and stimulating chemicals like caffeine also inhibit AChE and improve memory and cognition. Again, we postulate that the same or other plant compounds could also yield similar, even more effective, treatments. Furthermore, novel compounds from the metabolism of these prosaic foods through bacterial or microorganism-derived modification may yield beneficial effects. For example, Hwangryunhaedok-tang (HT), an Asian herb, when fermented with *Lactobacillus* spp., has enhanced anti-inflammatory effects through suppression of mitogen-activated protein kinase signaling in LPS-stimulated RAW264.7 cells in vitro [61]. HT also exerts neuroprotective effects on memory impairment by reducing cholinergic system dysfunction and inflammatory response in vascular dementia in a rat model [62]. HT, as well as black tea, green tea, and coffee, contains compounds which inhibit acetylcholinesterase, but they may contain many other enzymes, such as thiaminases, which degrade thiamin(e) (vitamin B1), another important compound in brain health and energy homeostasis. It is transketolase and the diversion of glycolytic intermediates to the hexose monophosphate shunt that protect against glycation damage in diabetes and perhaps even type 3 diabetes [63] or AD. Moreover, green tea metabolites inhibit beta secretase and could prevent the release of Aβ peptides from APP. However, coffee is a less effective inhibitor of acetylcholinesterase, having no butyryl cholinesterase or beta secretase inhibitory activity [64]. Finally, Hwangryunhaedok-tang was shown to have anti-inflammatory, antiplatelet, and antithrombotic activity [65]. This herb is but one example of prosaic foods having activity after metabolism or bacterial fermentation processes.

## 7. Microorganisms Found in Alzheimer Disease

During the course of aging, the gastrointestinal tract epithelial barrier and the blood–brain barrier become significantly more permeable, which would make the CNS more susceptible to potential neurotoxins generated by microbiome-resident or environmental pathogens (see Figure 1). The contribution of pathogenic microbial populations to the progression of AD is being established and altered microbiome signaling from other disease-inducing agents, such as fungal infection of the CNS and viral infections, may contribute to the development of AD [3]. Chronic fungal infections and disseminated diffuse mycoses found in the peripheral blood of AD patients may help explain increase AD risk from the mycobiota. In that regard, yeast and fungal proteins like (1,3)-β-glucan or high levels of fungal polysaccharides are associated with increased AD risk [66]. A number of viral agents, common viruses, and latent viral infections can establish lifelong latency in the CNS, and they are linked to the development of AD. For example, *Herpesviridae* and *Herpes simplex virus-1* (HSV-1), human cytomegalovirus, and hepatitis C infections were shown to significantly increase the risk of AD, especially in the elderly [3]. *Chlamydophila pneumoniae* is another pathogenic Gram-negative, obligate intracellular bacteria associated with AD and coronary artery disease [67]. Chagas disease caused by the protozoan *Trypanosoma cruzi* mainly affects the nervous system, digestive system, and heart. Enteric nervous system impairment caused by *T. cruzi* infection also has an association with *Helicobacter pylori* infection [68]. Toxoplasma species such as several intracellular protozoan parasites, specifically, *Toxoplasma gondii*, cross the BBB to cause neurological dysfunction and encephalitis by promoting chronic inflammation of the brain, meninges, and CNS. Moreover, AD is associated with significantly increased anti-*T. gondii* antibodies, which suggests a possible mechanistic link between *T. gondii* infection and AD via household pets [3,69].

Interestingly, red wine constituents may reverse some of the action of pathogens in various models. In Chagas disease, for example, resveratrol in mice was effective in reversing functional heart disease [70], and heart function was improved by sirtuin 1 (SIRT-1) targeted therapy for *Trypanosoma cruzi* infection [71]. In neuronal progenitor cells, resveratrol mediated a reversal in purinergic signaling and toxoplasma *T. gondii* immune responses [72]. Antibiotics, such as ofloxacin or clarithromycin, together with resveratrol or quercetin, showed inhibition and lowering of IL-23 levels, which suggested that the combined treatment might show a synergistic effect when controlling chlamydia infection [73]. Even viruses are potential treatment targets. For example, polyphenol-rich extract from berries, with high chlorogenic acid and delphinidin-3-rutinoside content, inhibited HSV-1 replication, while demonstrating scavenging and chelating properties, decreasing ROS production, and counteracting the catalase suicide molecule *t*-butyl hydroperoxide-induced cytotoxicity [74]. Moreover, the extract from the peel also increased intracellular glutathione levels, which suggests a possible hormetic mechanism of action.

Taken together, it is clear that the human CNS is under constant assault from a wide array of intrinsic and extrinsic neuroactive products from microbes, pathogens, viruses, and fungi found in the environment and contained within the microbiome. When we couple treatment with select polyphenols, many of the AD-implicated organisms are either controlled or respond to those administered polyphenols. Virtually every type of microbe known could be implicated in contributing to AD susceptibility and pathogenesis, which is especially true when blood immune barriers are breached [25]. This could be especially important with aging, because the innate immune system and physiological barriers are often compromised with advancing age [75], which would enable microbes and any neurotoxic metabolites produced to readily gain access to the CNS or other brain compartment. Since AD is clearly a multifactorial disease, having multiple biological pathways through which vulnerable neurons elicit dysfunction, it is not surprising that multiple and complex microbial insults contribute to AD, which include pathological signal transmission throughout the vagus nerves to the CNS.

From Part I to Part II, we come full circle in describing aspects of the French paradox and the MGB axis–heart shunt. In Part I of this series, we described the metabolism of foods and carnitine by gut microbes. In particular, we explored the enzymatic action of anaerobic bacteria and discussed dietary sources for TMANO, from choline, phosphatidylcholine (PC), and betaine, all of which produce trimethylamine-*N*-oxide [55] in Part I. In Part II, we explored gut metabolites and TMANO in possible AD pathogenesis and protection. We and others identified TMANO in human tissues and fluids including human cerebrospinal fluid and rodent brains. It was shown that TMANO lowers the critical concentration of tubulin needed for tubulin assembly by modulating binding of protein kinase A-phosphorylated microtubule-associated protein tau to form tubulin. It seems that TMANO levels are not related to neurological disorders [76]. However, since one hallmark of AD is hyper-phosphorylation of the microtubule-associated protein Tau, this finding offers a therapeutic implication for TMANO in Alzheimer disease. In support of this notion, it was found that TMANO not only affects the promotion, but also enhances the assembly of microtubules, as found experimentally with both mutant and hyper-phosphorylated tau protein [77]. TMANO does not act by dephosphorylating tau protein; rather, it facilitates the binding of the microtubule-associated protein tau and tubulin by reducing the critical concentration of tubulin necessary for assembly [78]. Furthermore, TMANO stabilizes and modifies the aggregation of Aβ peptide, and, with chaperone-like activity, it can prevent the conversion of Aβ peptide to its β-conformation and stabilize the resulting proto-fibrils, which might prevent aggregation and tangles [79]. TMANO may have a role in protecting the brain as discussed in Part I of this two-part series. This makes a case for the neurotransmitter acetylcholine and PC and choline in brain health, and the adequate intake of choline reduced the risk of dementia as it was observed that intake, mainly from eggs and meat, was associated with reduced dementia risk [80].

## 8. Conclusions

In summation, it is not clear after knowing what we now know about TMANO production, even from healthy foods with choline derivatives, how this could synergize with wine and its betaine and alcohol content to offer an explanation for the French paradox. Moreover, how the French and their well-known consumption of largely fatty, saturated, rich, and unhealthy foods (not unlike Americans) can actually have less heart disease and less AD than any other group is intriguing. Some suggest that smaller portions, chewing more, and less overall life stress could also help to explain differences. We suggest that bugs could act as drugs or psychobiotics, which implies that our microbiota and the right diet or foods may be all we need to affect many aspects of our mental, brain, and heart health.

Therefore, these findings demand that we explore the microbiota of the French and the co-metabolism of paradoxical foods, maybe even coupled with red wine polyphenols, in rodent and culture models. In that way, we may elucidate a mechanism and advance our understanding of how prosaic foods, in the right combination or with wine polyphenolic acids, could serve to prevent intractable neuronal disease. Perhaps, one day, we will consume a designer symbiotic and, thus, have our cake and eat it too, and, if Marie Antoinette were extant, she might rephrase her famous quote to say “Qu’ils mangent lentement et qu’ils mangent de la brioche, mais qu’ils n’oublient pas du vin!” (translation: “Let them eat slowly and let them eat cake, but let them not forget some wine!”); however, until such time, “à votre santé!” (translation: “to your health!”).

## Abbreviations

hypothalamic–pituitary–adrenal	HPA
trimethylamine-*N*-oxide	TMANO/TMAO
amyloid precursor protein	APP
interleukin	IL
tumor necrosis factor	TNF
endothelin-1	ET-1
reactive oxygen species	ROS
reactive nitrogen species	RNS
lipopolysaccharide	LPS
nonsteroidal anti-inflammatory drugs	NSAIDs
insulin-like growth factor-1	IGF-1
presenillin1	PS1
amyloid beta	Aβ
peroxynitrite	ONOO
nitric oxide	NO
inducible nitric oxide	iNOS
microbiota-gut-brain	MGB
celiac disease	CD
apolipoprotein E	ApoE4
acetyl-l-Carnitine	ALCAR
*R*-alpha-lipoic acid	LA
vascular dementia	VaD
hydrogen sulfide gas	H_2_S
cholesterol acyltransferase	ACAT
Hwangryunhaedok-tang	HT
small guanosine triphosphate	GTP
G-protein coupled receptor kinase	GRK
G-protein coupled receptor	GPCR
amyloid-derived diffusible ligands	ADDLS
acetylcholine	ACh
acetylcholinesterase	AChE
butyryl cholinesterase	BChE
oxidation–reduction or redox potential	Eh
herpes simplex virus-1	HSV-1
phosphatidylcholine	PC
